# A novel tomato interspecific (*Solanum lycopersicum* var. *cerasiforme* and *Solanum pimpinellifolium*) MAGIC population facilitates trait association and candidate gene discovery in untapped exotic germplasm

**DOI:** 10.1093/hr/uhae154

**Published:** 2024-06-03

**Authors:** Andrea Arrones, Oussama Antar, Leandro Pereira-Dias, Andrea Solana, Paola Ferrante, Giuseppe Aprea, Mariola Plazas, Jaime Prohens, María José Díez, Giovanni Giuliano, Pietro Gramazio, Santiago Vilanova

**Affiliations:** Instituto de Conservación y Mejora de la Agrodiversidad Valenciana, Universitat Politècnica de València, Camino de Vera 14, 46022 Valencia, Spain; Instituto de Conservación y Mejora de la Agrodiversidad Valenciana, Universitat Politècnica de València, Camino de Vera 14, 46022 Valencia, Spain; Instituto de Conservación y Mejora de la Agrodiversidad Valenciana, Universitat Politècnica de València, Camino de Vera 14, 46022 Valencia, Spain; Instituto de Conservación y Mejora de la Agrodiversidad Valenciana, Universitat Politècnica de València, Camino de Vera 14, 46022 Valencia, Spain; Agenzia Nazionale Per Le Nuove Tecnologie, L’energia e Lo Sviluppo Economico Sostenibile (ENEA), Casaccia Research Centre, Via Anguillarese 301, 00123 Rome, Italy; Agenzia Nazionale Per Le Nuove Tecnologie, L’energia e Lo Sviluppo Economico Sostenibile (ENEA), Casaccia Research Centre, Via Anguillarese 301, 00123 Rome, Italy; Instituto de Conservación y Mejora de la Agrodiversidad Valenciana, Universitat Politècnica de València, Camino de Vera 14, 46022 Valencia, Spain; Instituto de Conservación y Mejora de la Agrodiversidad Valenciana, Universitat Politècnica de València, Camino de Vera 14, 46022 Valencia, Spain; Instituto de Conservación y Mejora de la Agrodiversidad Valenciana, Universitat Politècnica de València, Camino de Vera 14, 46022 Valencia, Spain; Agenzia Nazionale Per Le Nuove Tecnologie, L’energia e Lo Sviluppo Economico Sostenibile (ENEA), Casaccia Research Centre, Via Anguillarese 301, 00123 Rome, Italy; Instituto de Conservación y Mejora de la Agrodiversidad Valenciana, Universitat Politècnica de València, Camino de Vera 14, 46022 Valencia, Spain; Instituto de Conservación y Mejora de la Agrodiversidad Valenciana, Universitat Politècnica de València, Camino de Vera 14, 46022 Valencia, Spain

## Abstract

We developed a novel eight-way tomato multiparental advanced generation intercross (MAGIC) population to improve the accessibility of tomato relatives genetic resources to geneticists and breeders. The interspecific tomato MAGIC population (ToMAGIC) was obtained by intercrossing four accessions each of *Solanum lycopersicum* var. *cerasiforme* and *Solanum pimpinellifolium*, which are the weedy relative and the ancestor of cultivated tomato, respectively. The eight exotic ToMAGIC founders were selected based on a representation of the genetic diversity and geographical distribution of the two taxa. The resulting MAGIC population comprises 354 lines, which were genotyped using a new 12k tomato single primer enrichment technology panel and yielded 6488 high-quality single-nucleotide polymorphism (SNPs). The genotyping data revealed a high degree of homozygosity, an absence of genetic structure, and a balanced representation of the founder genomes. To evaluate the potential of the ToMAGIC population, a proof of concept was conducted by phenotyping it for fruit size, plant pigmentation, leaf morphology, and earliness. Genome-wide association studies identified strong associations for the studied traits, pinpointing both previously identified and novel candidate genes near or within the linkage disequilibrium blocks. Domesticated alleles for fruit size were recessive and were found, at low frequencies, in wild/ancestral populations. Our findings demonstrate that the newly developed ToMAGIC population is a valuable resource for genetic research in tomato, offering significant potential for identifying new genes that govern key traits in tomato. ToMAGIC lines displaying a pyramiding of traits of interest could have direct applicability for integration into breeding pipelines providing untapped variation for tomato breeding.

## Introduction

Tomato (*Solanum lycopersicum* L.) is the most economically important vegetable crop and a model plant species, with an extensive pool of genetic tools and resources. The tomato research community has access to a wealth of genetic information for wild species, landraces, and modern cultivars, including high-quality genome sequences [1]. Several databases compiling genomic, genetic, transcriptomic, phenotypic, and taxonomic information are available [2–6]. Over decades, several tomato biparental populations have also been released including introgression lines, recombinant inbred lines, and advanced backcrosses, among others (e.g. [7–12]).

In the genomics era, new multiparental populations have been developed dramatically increasing mapping resolution [13]. Multiparental advanced generation intercross (MAGIC) populations are powerful next-generation prebreeding resources with increased diversity and high recombination rates, suitable for quantitative trait locus (QTL) mapping and candidate gene identification [13–16]. In tomato, only two MAGIC populations have previously been released. The first one was a MAGIC population developed by crossing four large-fruited *S. lycopersicum* accessions with four cherry-type accessions of *S. lycopersicum* var. *cerasiforme* (SLC) [17]. Final lines were used to study fruit weight distribution in the population in different environments, identifying QTLs that colocalized with already cloned genes. Subsequently, Campanelli *et al.* [18] developed a MAGIC population that included seven cultivated accessions of tomato and one of the wild *Solanum cheesmaniae* as founders. The *S. cheesmaniae* accession was selected for its biotic and abiotic stress tolerance, yield, and resiliency[19].

The development of MAGIC populations using wild species as founders represents a promising way to combine the potential of these experimental populations for QTL/gene mapping together with the exploitation of the large phenotypic and genetic variation from the wild donor introgressions. Here, we present a novel eight-way interspecific tomato MAGIC population (ToMAGIC) obtained by using SLC and *Solanum pimpinellifolium* (SP) accessions as founders, which are the closest relative and the ancestor of cultivated tomato, respectively [20]. Cultivated tomato suffered strong genetic bottlenecks during domestication and breeding processes, resulting in low genetic diversity of tomato landraces and heirlooms [21]. Based on previous morphological characterization and resequencing data availability, the eight selected founders of the new ToMAGIC population represent a wide genetic and morphological variation, as well as differences in ecological adaptation [21, 22]. Founders are very diverse in terms of not only fruit, vegetative, and flowering traits but also their capacity of adaptation to different conditions, ranging from desert to tropical forest environments and from sea level to over 1500-m altitude. Therefore, one of the aims of this population is to recover Andean variability lost during the domestication process by using a substantial proportion of the fully cross-compatible weedy and wild tomato diversity.

This ToMAGIC population may have a large potential to identify new genomic regions and candidate genes of interest in breeding, as well as to validate genes and QTLs already described in a genetic background other than that of cultivated tomato. In this way, another aim of this population is dissecting the control of different traits, including those involved in the early domestication of tomato [23]. The introduction of exotic germplasm will be useful for shedding light on the genetics of agronomic and adaptation traits present in these materials, as well as for the selection of elite lines of interest for tomato breeding [14]. In our work, the integration of high-throughput genotyping of the recombinant ToMAGIC population together with the phenotyping of specific traits across different plant parts has effectively demonstrated a proof of concept for the high-precision fine mapping of these traits. This approach has not only validated previously identified candidate genes for the traits studied in an SLC and SP genetic background but also led to the discovery of new candidate genes and the observation of additional phenotypic-causing variants, underscoring the great potential of the ToMAGIC population for tomato genetics and breeding.

## Results

### MAGIC population construction

**Figure 1:**
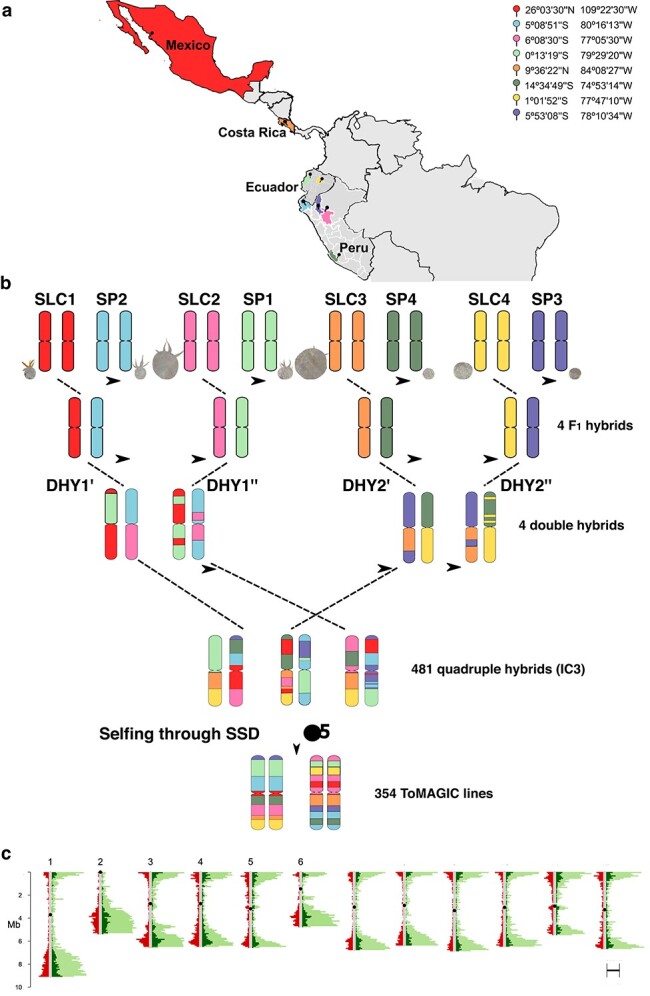
(A) Origin of the different SLC and SP founders selected for the ToMAGIC population development represented with the different colour codes. (B) The funnel breeding design to develop the 354 ToMAGIC lines. The eight founders with a different colour to represent their genomic background are represented at a scale based on the real fruit size. Arrows indicate the direction of the cross. (C) Distribution of the 6488 filtered markers (in red), the Heinz 1706 SL4.0 annotated genes (in light green), and the genes covered by the filtered markers (in dark green) across the 12 tomato chromosomes.

In the first stage of MAGIC population development, SLC and SP accessions of different origins (Fig. 1A) were intercrossed pairwise (Fig. 1B). These materials are native to different geographic regions of South and Central America, mainly from Ecuador and Northern Peru, and provide a representation of the Andean variability lost during the domestication process in Mesoamerica (Fig. 1A). They were selected since they are considered genetic diversity reservoirs barely exploited in tomato breeding [22]. They include a wide molecular variability and phenotypic diversity in plant and inflorescence architecture, leaf, flower, and fruit traits, together with resistance or tolerance (in some of the founders) to biotic and abiotic stresses [21], including water and salt stress adaptation [24]. The eight founders have previously been characterized morphoagronomically and their genomes have been resequenced [21, 22].

These weedy (SLC) and wild (SP) tomato species are cross-compatible [22], and thus the manual intercross was successfully performed. As a result of the intercross of the eight founders, the F_1_ hybrids, and the DHY hybrids, 112 IC1 individuals were obtained. The subsequent intercrossing following a chain pollination scheme resulted in the obtention of 232 IC2 and 481 IC3 individuals. The latter individuals were self-pollinated to produce 475 S1, 452 S2, 427 S3, 400 S4, and the final population of 354 S5 (ToMAGIC) lines (Fig. 1B).

### Genotyping

A total of 4 268 587 SNPs were generated from the genotyping of the 354 ToMAGIC lines using a newly developed 12k probes tomato single primer enrichment technology (SPET) panel (Aprea *et al*., in preparation). After filtering, 6488 markers were retained for the subsequent genome-wide association study (GWAS) analysis. A higher marker density was observed in gene-rich regions located in distal chromosomal regions (Fig. 1C). The distribution of SNPs among the different tomato chromosomes was fairly uniform, with an average marker density of 8.51 per Mb (Table 1). This ratio is an average of the whole chromosome including the centromere where recombination and gene density are extremely low as observed in Fig. 1C. Excluding centromeric regions, marker density in the euchromatic regions increased to 17.95 markers per Mb, which is equivalent to almost two markers per 100 kb. The filtered markers cover 16.91% of the total annotated genes. The residual heterozygosity of the ToMAGIC lines was on average 6.31%.

**Table 1:** Chromosome-wide distribution of the SNP positions used for the GWAS in the tomato MAGIC population.

**Chromosome**	**Markers**	**% Markers**	**Chromosome length (Mb)**	**Marker density (markers/Mb)**	**Marker density in euchromatic regions (markers/Mb)**	**Genes**	**Covered genes**
1	646	9.96	90.86	7.11	18.15	4.133	619
2	568	8.75	53.47	10.62	20.58	3.379	518
3	624	9.62	65.30	9.56	20.37	3.324	578
4	815	12.56	64.46	12.64	23.15	2.819	689
5	656	10.11	65.27	10.05	17.61	2.382	496
6	408	6.29	47.26	8.63	16.22	2.769	392
7	425	6.55	67.88	6.26	14.22	2.517	379
8	346	5.33	64.00	5.41	12.32	2.428	315
9	436	6.72	68.51	6.36	17.87	2.521	403
10	406	6.26	64.79	6.27	14.11	2.520	342
11	552	8.51	54.38	10.15	19.31	2.326	446
12	606	9.34	66.69	9.09	21.48	2.444	498
Total	6.488	100	772.87			33.562	5.675
Average		8.33		8.51	17.95		

### Population structure

**Figure 2:**
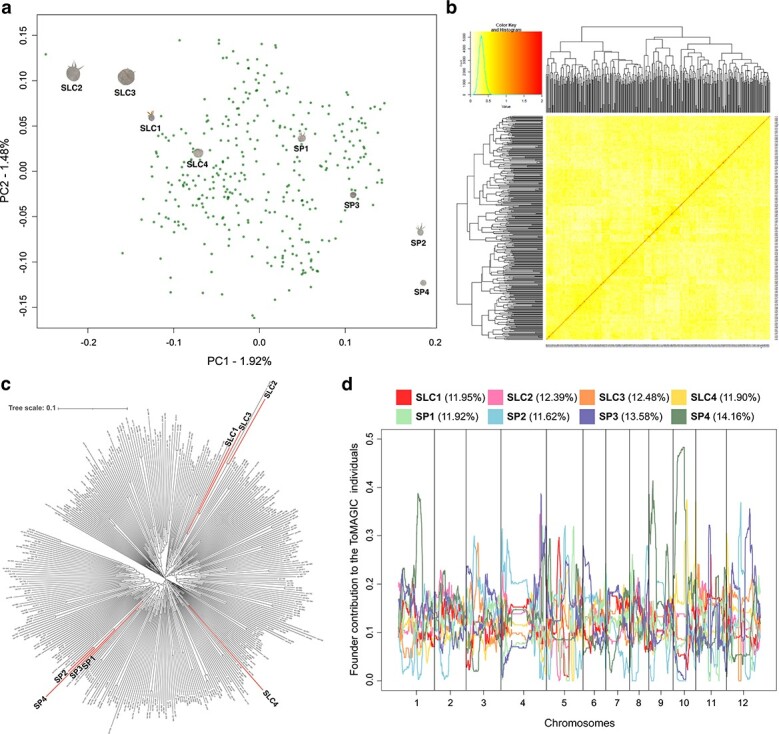
Population structure of the interspecific ToMAGIC population. (A) PCA plot of the first two PCs. (B) Heat map plot of genetic relationship based on the kinship matrix. (C) Dendrogram indicating founders’ locations with coloured red branches. (D) Genome-wide founder haplotype blocks assignment across the 12 tomato chromosomes (*x*-axis) as the average percentage of founders’ contribution to the ToMAGIC lines (*y*-axis) with a different colour associated with each founder.

A lack of genetic structure in ToMAGIC population was supported by the principal component analysis (PCA), in which no differentiated groups were observed (Fig. 2A). The first two principal components (PCs) accounted only for 3.40% of the genetic variance, whereas the first 10 PCs, 9.93%, and it required 41 PCs to explain 20% of the genetic variation, underscoring the weak population structure of the population. In addition, kinship coefficients between pairs of ToMAGIC lines varied from 0 to 1.32 (on a scale of 0 to 2), with 98.35% of the pairs with kinship values <0.5 (Fig. 2B). These results revealed a low genetic relatedness among ToMAGIC lines.

SLC founders were grouped close together, with negative values of the PC1, while SP founders had positive values for the PC1 (Fig. 2A). A similar grouping was observed in the dendrogram of the MAGIC population and founders (Fig. 2C). SLC2 and SLC3 are the closest accessions to cultivated tomato and plot in the first PCA quadrant with low values for the PC1 and high for the PC2. SLC4 is the closest to SP founders in the PCA (Fig. 2A) and is separated from the rest of SLC founders in the dendrogram (Fig. 2C). The estimated average contribution of each founder to the overall population was around the theoretically expected value of 12.50%, with the range varying from 11.62% for SP2 to 14.16% for SP4. However, the reconstruction of genome mosaics for the 354 ToMAGIC lines, considering the eight founder haplotypes, revealed different haplotype block proportions at different chromosomal positions (Fig. 2D).

### Phenotyping analysis

Phenotyping for locule number, fruit weight, plant anthocyanin pigmentation, leaf lobing/serration, leaf complexity, and number of leaves below the first inflorescence revealed a wide range of variation, including transgressive lines for some of the studied traits (Table 2, Fig. S1). For the locule number trait, the average for SP founders was 2 locules, while the average for SLC was 2.75 and the range between 2 and 4. However, ToMAGIC lines with up to 5 and 6 locules were identified, although most of the lines only had 2 locules, resulting in an average value of 2.2. For the fruit weight, ToMAGIC lines showed an intermediate average (2.72 g) between the SP and SLC founders weight averages of 1.60 and 4.97 g, respectively. However, the range of variation of the founders was greater (from 0.97 to 11.59 g) than those of the ToMAGIC lines (0.44 to 7.01), and no lines were found with a higher weight than the heaviest founder (SLC3). For the plant anthocyanin pigmentation, the mean of SLC founders (0.50) was lower than that of the SP founders (1.25), mainly due to the high level of plant pigmentation of the SP4 founder. The range of variation was greater for the ToMAGIC lines (from 0 to 4) than for the founders (from 0 to 3). For the leaf lobing/serration, ToMAGIC lines showed an intermediate average (3.69 g) between the SP and SLC founders averages of 2.50 and 6, respectively. The ToMAGIC lines covered all the variation range found in the founders, from the lack of lobing/serration (1) to very serrated leaves (7). For the leaf complexity, ToMAGIC lines showed an intermediate average (0.26) between the SP (0) and SLC (0.50) founders. For the number of leaves below the first inflorescence, the SP founders had a slightly lower number (4.33) than SLC founders (6.66), while ToMAGIC lines had an average of 5.36 leaves. However, the range of variation was much larger for the ToMAGIC lines (from 4 to 10) than for the founders (from 4 to 7). Pearson pairwise correlations among the traits evaluation were conducted, and only a slight positive correlation (*r* = 0.3261; *P* = 1.57e^−7^) between leaf lobing/serration and leaf complexity was observed (Fig. S2B).

**Table 2:** Means and range values for SLC and SP founders, and ToMAGIC lines for the phenotypic traits evaluated.

**Trait**	**SLC**		**SP**		**ToMAGIC lines**	
	**Average**	**Range**	**Average**	**Range**	**Average**	**Range**
Locule number	2.75	2–4	2	2	2.20	2–6
Fruit weight (g)	4.97	1.61–11.59	1.60	0.97–2.89	2.72	0.44–7.01
Plant anthocyanin	0.50	0–1	1.25	0–3	0.94	0–4
Leaf lobing/serration	6	5–7	2.50	1–3	3.69	1–7
Leaf complexity	0.50	0–1	0	0	0.26	0–1
Number of leaves below the first inflorescence	6.66	6–7	4.33	4–5	5.36	4–10

### Fruit size

#### Locule number

**Figure 3:**
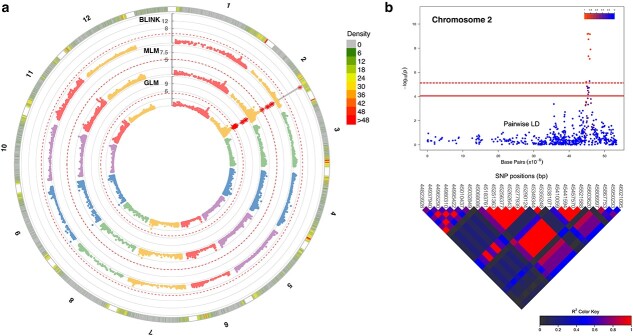
Genome-wide association results for the locule number trait. (A) Manhattan plots comparing GLM, MLM, and BLINK models. The solid grey line indicates the common significant markers detected by two or more models. The red asterisks indicate the SNPs exceeding the Bonferroni threshold, represented as a dashed red line. (B) On the top, a chromosome-wise Manhattan plot with the top significant markers. Bonferroni and FDR thresholds are represented with red dashed and continuous lines, respectively. The colour from blue to red indicates *r*^2^ from 0 to 1. On the bottom, heat map of pairwise LD. SNP positions under the significant region are indicated in bp. The colour from black to red indicates *r*^2^ from 0 to 1.

The Manhattan plot for fruit locule number revealed one significant peak on chromosome 2 (Fig. 3A, Table 3). For the GLM model, 28 SNPs were above the false discovery rate (FDR) threshold (logarithm of odds (LOD) > 3.73), 20 of them over the Bonferroni threshold (LOD > 5.11) between 44.78 and 46.13 Mb. For the MLM model, 16 SNPs were above the FDR threshold (LOD > 4.19), nine of them over the Bonferroni threshold between a reduced region of 44.82 and 46.02 Mb (Fig. 3B). For the BLINK model, a single SNP was above the FDR (LOD > 15.27) and Bonferroni thresholds at the 45.87-Mb position. This association peak accounted for 26.84% of the total phenotypic variance of the locule number trait.

**Table 3:** Association analysis results for GLM, MLM, and BLINK models and list of candidate genes for locule number, fruit weight, plant anthocyanin, leaf lobing/serration, leaf complexity, and number of leaves below the first inflorescence.

**Trait**	**GLM**			**MLM**			**BLINK**			**Candidate genes**		
	**Chromosome**	**Genomic region (Mb)**	**LOD**	**Chromosome**	**Genomic region (Mb)**	**LOD**	**Chromosome**	**Genomic region (Mb)**	**LOD**	**Abbreviation**	**Name**	**Position (bp)**
Locule number	2	44.78–46.13	11.39	2	44.82–46.02	9.20	2	45.87	15.27	*WUSCHEL*	Solyc02g083950.3.1	45 191 157–45 192 582
Fruit weight	2	50.51–50.55	5.37	-	-	-	2	50.55	5.33	*FW2.2*	Solyc02g090730.3.1	50 292 691–50 293 481
Plant anthocyanin	7	8.38–61.70	15.28	7	59.97–60.88	12.42	7	60.44	21.14	*SlMYB-ATV*	Solyc07g052490.4.1	60 912 702–60 913 855
	2	27.13–33.38	5.14	-	-	-	2	33.38–46.91	7.64	*bHLH*	Solyc02g063430.4.1	33 546 773–33 549 186
Leaf lobing/serration	4	62.30–63.23	11.63	4	62.30–62.91	9.51	4	62.87	6.46	*AP3/DEF*	Solyc04g081000.3.1	63 032 681–63 036 255
										*OVATE9*	Solyc04g080210.1.1	62 437 899–62 438 699
										*ANT*	Solyc04g077490.3.1	60 418 478–60 421 941
Leaf complexity	4	62.49–62.73	5.84	4	62.49	5.38	4	62.49	8.93	*KNOTTED1*	Solyc04g077210.3.1	60 124 504–60 131 770
										*IAA9*	Solyc04g076850.3.1	59 750 087–59 755 552
Number of leaves below the first inflorescence	11	2.05–2.80	9.19	11	2.17–2.80	8.54	11	2.80	24.22	*FT1*	Solyc11g008640.1.1	2 854 837–2 857 237
										*FT2*	Solyc11g008650.1.1	2 866 945–2 867 166
										*SP1*	Solyc11g007880.1.1	2 135 303–2 135 602
										*J*	Solyc11g010570.2.1	3 671 232–3 676 350

In the genomic candidate region on chromosome 2, the *WUSCHEL* gene (Solyc02g083950.3.1, 45 191 157–45 192 582 bp) was identified (Table 3). *WUSCHEL* gene controls stem cell fate in the apical meristem directly affecting locule number during tomato fruit development [25, 26]. The two multilocular founders of the ToMAGIC population, SLC2 and SLC3, showed two SNPs immediately downstream of the *WUSCHEL* gene that were previously described as directly associated with an increased locule number [26]. Specifically, a T/C transition at 45189386 bp and an A/G transition at 45189392 bp are considered as the responsible SNPs for the locule number trait (Fig. S2A). These two SNPs were in almost complete linkage disequilibrium (LD), and they are considered as a unique haplotype.

**Figure 4:**
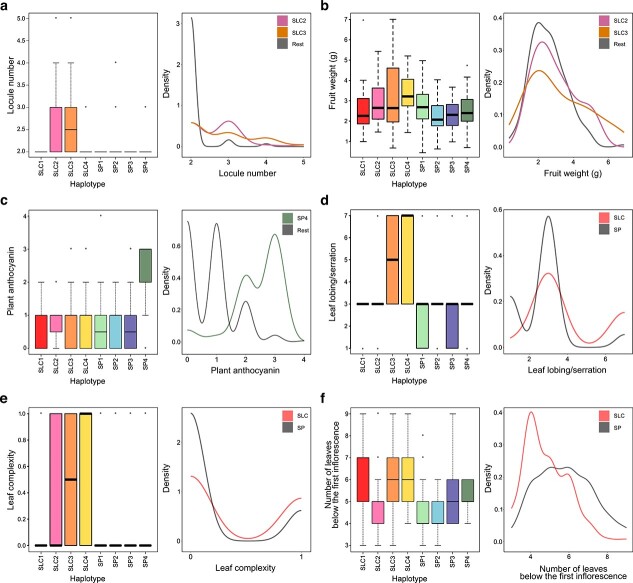
Haplotype analysis of the ToMAGIC lines for each of the MAGIC founders’ haplotype in combination with phenotypic data. Boxplot and density plot distribution in the candidate genomic regions for (A) locule number, (B) fruit weight, (C) plant anthocyanin on chromosome 7, (D) leaf lobing/serration, (E) leaf complexity, and (F) number of leaves below the first inflorescence. Boxplots represent the ToMAGIC lines phenotypes associated with the eight haplotypes with the different colour codes for each founder, and hollow dots correspond to outliers. Density plots represent the variation among groups that show significant differences.

Haplotype analyses were performed to associate the candidate genomic regions with the phenotypic effects. For the locule number, a significant difference was observed between the haplotype of the SLC2 and SLC3 founders, which are the ones showing more than 2 locules, and the rest of the haplotypes of the ToMAGIC founders according to pairwise *t*-test for multiple comparisons (Fig. 4A). When generating the density plot, higher values were also associated with the SLC2 (at 3 locules) and SLC3 (at 4 locules) founder haplotype. The density curve of the rest of the founder haplotypes exceeds density values of 1, since fruits with only two locules predominate in the ToMAGIC population.

#### Fruit weight

The Manhattan plot for fruit weight also revealed one significant peak on chromosome 2, although only for GLM and BLINK models (Fig. S3A, Table 3). For the GLM model, eight SNPs were above the FDR threshold (LOD > 4.15), three of them over the Bonferroni threshold (LOD > 5.11) between 50.51 and 50.55 Mb (Fig. S3B). For the BLINK model, a single SNP was above the FDR (LOD = 5.33) and Bonferroni thresholds at 50.55 Mb position. This association peak explained 14.76% of the total phenotypic variance of the fruit weight trait.

Under the significant peak on chromosome 2, the well-known *FW2.2* gene (Solyc02g090730.3.1, 50 292 691–50 293 481 bp) was identified (Table 3). This gene is differentially expressed in floral development and controls carpel cell division [27]. The wild-type SNP was identified in all the ToMAGIC founders, except for founders SLC2 and SLC3, which have larger fruit weights [21]. This SNP corresponds to a C/T change upstream of the 5′ region of *FW2.2* gene at 50292019 bp (Fig. S2A).

In the haplotype analysis, pairwise *t*-test revealed a significant difference between SLC2 and SLC3 on one side and SP founders from the other (Fig. 4B). When generating the density plot, most of the lines are around 2 to 3 g since light fruits predominate in the ToMAGIC population with an average weight of 2.72 g (Table 2). Lines with weights greater than 3 show mostly SLC2 and SLC3 haplotypes.

### Plant pigmentation

The Manhattan plot for plant anthocyanin revealed two significant peaks: one major peak on chromosome 7 and one minor but significant peak on chromosome 2 (Fig. S4A, Table 3). For the GLM model, 43 SNPs were above the FDR threshold (LOD > 3.33) on chromosome 7, 21 of them over the Bonferroni threshold (LOD > 5.11) between 8.38 and 61.70 Mb. On chromosome 2, 14 SNPs were above the FDR threshold, being only two of them over the Bonferroni threshold between 27.13 and 33.38 Mb. For the MLM model, only one association peak was identified on chromosome 7 with eight SNPs over the FDR threshold (LOD > 4.17), five of them over the Bonferroni threshold between a reduced region of 59.97 and 60.88 Mb (Fig. S4B). For the BLINK model, a single SNP was above the FDR (LOD > 5.67) and Bonferroni thresholds reaching an LOD of 21.14 on chromosome 7 at 60.44 Mb position. On chromosome 2, only two SNPs were above the FDR and Bonferroni thresholds at 33.38 and 46.91 Mb positions reaching an LOD of 7.64 and 6.70, respectively. The association peak on chromosome 7 explained 15.14% of the total phenotypic variance of the plant anthocyanin trait, while the peak on chromosome 2 explained 4.68% of the phenotypic variance.

Under the major GWAS peak on chromosome 7, in the genomic region of 60 912 702–60 913 855 bp, a *MYB-like* transcription factor (*SlMYB-ATV,* Solyc07g052490.4.1) was identified (Table 3). The *SlMYB-ATV* (myeloblastosis–atroviolacea) gene has been described as a repressor of anthocyanin synthesis in vegetative tissues of tomato plants [28]. However, we did not observe the previously described mutations in the gene sequence in our accessions. A 9-base pair in-frame deletion at the 60,912,903 bp position, deleting 3 amino acids in the transcriptional repressor MYB domain, was identified in the SP4 founder. This founder is unique for displaying anthocyanins in all plant parts (Fig. S2A).

The same procedure was followed for the minor peak on chromosome 2. All the genes located near or within the LD block were assessed by SnpEff [29] for all of the MAGIC founders. We found the *bHLH* transcription factor (Solyc02g063430.4.1 between 33 546 773 and 33 549 186), which belongs to a family involved in the regulation of anthocyanin biosynthesis in plants [30] (Table 3). However, no high-effect variants were predicted distinguishing between anthocyanin-containing and anthocyaninless founders.

In the haplotype analysis for chromosome 7, a significant difference was observed between the SP4 founder, which is the one showing increased levels of plant anthocyanins, and the rest of the ToMAGIC founders according to pairwise *t*-test (Fig. 4C). When generating the density plot, higher anthocyanin values were also associated with the SP4 founder haplotype.

### Leaf morphology

#### Leaf lobing/serration

The Manhattan plot for leaf lobing/serration revealed one significant peak on chromosome 4 (Fig. S5A, Table 3). For the GLM model, 20 SNPs were above the FDR threshold (LOD > 3.86), 10 of them over the Bonferroni threshold (LOD > 5.11) between 62.30 and 63.23 Mb. For the MLM model, nine SNPs were above the FDR threshold (LOD > 4.72), and nine of them over the Bonferroni threshold between a reduced region of 62.30 and 62.91 Mb (Fig. S5B). For the BLINK model, a single SNP was above the FDR (LOD > 6.46) and Bonferroni thresholds at 62.87 Mb position. This association peak accounted for 53.84% of the total phenotypic variance of the leaf lobing/serration trait.

Different genes involved in the leaf shape were detected within the candidate genomic region on chromosome 4 identified in the GWAS for the leaf lobing/serration (Table 3). In order of proximity to the candidate region, we found the *APETALA3/DEFICIENS* or *AP3/DEF* gene (Solyc04g081000.3.1 between 63 032 681 and 63 036 255 bp), which has been described as a regulator of petal and sepal development [31], the ovate family protein 9 or *OVATE9* gene (Solyc04g080210.1.1 between 62 437 899 and 62 438 699 bp), which belongs to a family protein that regulates different plant organs shape, including cotyledons, leaves, and fruits [32], and the *AP2-like* ethylene-responsive transcription factor *AINTEGUMENTA* or *ANT* gene (Solyc04g077490.3.1 between 60 418 478 and 60 421 941 bp), which plays a role as an auxin regulator in shoot and flower meristem maintenance, organ size and polarity, flower initiation, ovule development, floral organ identity, and cell proliferation [33]. No high-effect variants were predicted by SnpEff in the coding sequence of these genes contrasting for the different founders’ phenotypes.

Haplotype results revealed a significant difference between SLC and SP founders according to pairwise *t*-test (Fig. 4D). Although the haplotypes density plot also did not show a bimodal distribution for SLC and SP founders, it showed a higher density for SP haplotypes in lines exhibiting lack of lobing/serration or moderate lobing values, and a slightly higher density for SLC haplotypes in the very serrated leaf values.

#### Leaf complexity

The Manhattan plot for leaf complexity revealed one significant peak on chromosome 4 (Fig. S6A, Table 3). For the GLM model, three SNPs were above the FDR threshold (LOD > 4.70), two of them over the Bonferroni threshold (LOD > 5.11) between 62.49 and 62.73 Mb (Fig. S6B). For the MLM model, a single SNP was above the FDR (LOD > 5.38) and Bonferroni thresholds at 62.49 Mb position. At the same position for the BLINK model, a single SNP was above the FDR (LOD > 4.95) and Bonferroni thresholds reaching an LOD of 8.93. This association peak accounted for 4.12% of the total phenotypic variance of the leaf complexity trait.

Two genes involved in the leaf complexity were detected within the candidate genomic region on chromosome 4 identified in the GWAS (Table 3). In order of proximity to the candidate region, we found the *KNOTTED1* gene (Solyc04g077210.3.1 between 60 124 504 and 60 131 770 bp), which is expressed during leaf development and affects leaf morphology altering leaf complexity [34], and the *entire* or *INDOLE-3-ACETIC ACID9 IAA9* gene (Solyc04g076850.3.1 between 59 750 087 and 59 755 552 bp), which controls leaf morphology from compound to simple leaves [35]. No high-effect variants were predicted by SnpEff in the coding sequence of these genes for the founders with contrasting phenotypes.

Haplotype results revealed a significant difference between SLC and SP founders according to the pairwise *t*-test (Fig. 4E). Although the haplotypes density plot did not show a bimodal distribution for SLC and SP founders, it showed a higher density for SP haplotypes in pinnate leaves, and a slightly higher density for SLC haplotypes in the bipinnate leaves.

### Earliness

The Manhattan plot for the number of leaves below the first inflorescence revealed one significant peak on chromosome 11 (Fig. S7A, Table 3). For the GLM model, seven SNPs were above the FDR threshold (LOD > 4.30), four of them over the Bonferroni threshold (LOD > 5.11) between 2.05 and 2.80 Mb. For the MLM model, two SNPs were above the FDR (LOD > 5.53) and Bonferroni thresholds between a reduced region of 2.17 and 2.80 Mb (Fig. S7B). For the BLINK model, a single SNP was above the FDR (LOD > 4.95) and Bonferroni thresholds reaching an LOD of 24.22 at 2.80 Mb position. The association peak explained 5.52% of the total phenotypic variance of the number of leaves below the first inflorescence trait.

Different genes implicated in the flowering pathway were identified in the candidate genomic region on chromosome 11 proposed in the GWAS for the number of leaves below the first inflorescence (Table 3). In order of proximity to the candidate region, we found two *FLOWERING LOCUS T* (*FT*) genes (*FT1* Solyc11g008640.1.1 between 2 854 837 and 2 857 237 bp and *FT2* Solyc11g008650.1.1 between 2 866 945 and 2 867 166 bp), which have been described as mediating the onset of flowering and the floral transition in all angiosperms [36], the *SELF-PRUNING INTERACTING PROTEIN 1* or *SP1* gene (Solyc11g007880.1.1 between 2 135 303 and 2 135 602 bp), which is involved in a conserved signalling system that regulates flowering [37], and the *JOINTLESS* or *J* gene (Solyc11g010570.2.1 between 3 671 232 and 3 676 350 bp), which plays a role in flowering promotion [38]. The FT1 and FT2 proteins have a 71.68% (124/173) and 87.69% (57/65) identity, respectively, with the well-known *SINGLE-FLOWER TRUSS* (*SFT*, Solyc03g063100.2.1) gene product according to BLASTp alignment. While *FT1* is recognized as a paralogue of the *SFT* gene in EnsemblPlants, *FT2* seems to be a truncated pseudogene. Nevertheless, no clear variants were predicted by SnpEff in the coding sequence of these genes contrasting for the different founders’ phenotypes.

Haplotype results did not differentiate between SLC and SP founders (Fig. 4F). Pairwise *t*-test only revealed a significant difference between SLC1, SLC3, and SLC4 from SLC2, SP1, and SP2 founders, with SP3 and SP4 in intermediate positions. The haplotype density plot also did not show a bimodal distribution for SLC and SP founders. However, it showed a trend for lower number leaves below the first inflorescences for the SP haplotypes, while SLC haplotypes were distributed along a wide range of number of leaves below the first inflorescence.

## Discussion

We present a novel interspecific ToMAGIC population of 354 lines constructed by combining the genomes of SLC and SP founders. SLC accessions are phylogenetically positioned between SP and cultivated tomato [21, 39]. Therefore, founders were selected to exploit the wide diversity found in the tomato’s closest relatives taking advantage of their interbreeding compatibility [20]. Previous resequencing of the selected founders allowed to significantly enhance recombination detection, haplotype prediction, and causal variants identification within the MAGIC population [22].

The MAGIC population was generated through a systematic “funnel” approach [14] involving multiple rounds of intercross of the eight selected founders and five generations of selfing, totalling 10 generations. The three intercrossing generations from the two double hybrids and the blind single seed descent (SSD) process ensured high levels of recombination, maintaining a high genetic and morphological diversity. The final population consisted of 354 ToMAGIC lines, which was considered an appropriate population size to detect QTLs according to (i) tomato genome size [40], (ii) simulations of the power for detection of QTLs of an eight-way MAGIC population [41], and (iii) population size of previously developed tomato MAGIC populations [17, 18]. The ToMAGIC lines were genotyped by using a newly developed 12k probes tomato panel, based on SPET, which is a robust technology based on target SNPs, but also capable of discovering novel SNPs [42]. Although SPET has been mostly used in the biomedical field, it has demonstrated its potential as a high-throughput and high-efficiency genotyping platform in *Solanum* species [43, 44]. In this study, more than 4 million SNPs were generated with the 12k probes tomato SPET panel. After stringent filtering, 6488 were retained as markers, while in the previous tomato MAGIC population developed by Pascual *et al.* [17], 1486 markers obtained by a custom-made genotyping platform (Fluidigm 96.96 Dynamic Arrays, San Francisco, CA) were used for population analyses. In general, genetic diversity within the phylogenetic groups of the tomato clade is relatively low, which is one of the main reasons of the reduction in the final number of SNPs. Genomic divergence is estimated as 0.6% between SP and cultivated tomato, whereas most of SNPs are distributed in gene-poor regions [45]. The genotypic data revealed the absence of genetic structure, which is one of the advantages of MAGIC populations [14], and a balanced representation of the founder genomes. The average contribution of each founder to the overall population was around 12.50%, which is the expected value for a population developed from eight founders.

We have demonstrated the power of our ToMAGIC population for the fine mapping of traits of interest in tomato breeding. Specifically, GWAS analysis detected strong associations for all the traits evaluated using three different models (GLM, MLM, and BLINK), supporting the robustness of the associations detected [46–48]. This population could also be used to validate candidate genomic regions or genes previously identified through selective sweeps.

The implementation of SLC and SP accessions as founders has introduced a wide genetic and phenotypic diversity in the ToMAGIC population [21, 22]. Our proof of concept, focusing on a subset of traits from different plant parts, has revealed a large phenotypic diversity in the ToMAGIC population, including transgressive lines to some of the founders for all traits except leaf morphology. Within the phenotypic diversity of the final population, wild alleles showed a dominant effect over domesticated alleles in most traits. For instance, ToMAGIC lines tend to produce small fruits and simpler leaves, more similar to SP than to cultivated tomato. This prevalent dominance of wild alleles has been previously observed during the development of other interspecific populations [49].

Large tomato fruit size is a typical domestication trait, controlled by at least five different genes [50]. It is tempting to speculate that, similar to the nonshattering spike trait in cereals [51], it negatively affects plant fitness in the wild, by reducing seed dispersal by small vertebrates. Drawing on this parallel, the most likely scenario is that recessive alleles for large fruit size in tomato and nonshattering spike in cereals were both preexisting in wild/weedy populations, and that they were not completely counterselected due to their recessive nature. Under this scenario, human selection for higher harvestable biomass probably acted on the rare homozygous plants that appeared in these wild populations. Consistent with this hypothesis, the nonfunctional (domesticated) allele of the rice shattering gene *sh4* is found, at low frequency, in the wild ancestor *Oryza rufipogon* [52].

Almost all wild tomato species produce bilocular small fruits, and therefore, locule number and fruit weight played a crucial role in the increase in fruit size during domestication [25, 53, 54]. On one hand, as a result of the GWAS analysis for locule number, an associated genomic region was identified that colocalized with the *WUSCHEL* gene. Mutations on this gene have been necessary to increase locule number during domestication [26]. However, previous sequence analysis on this gene revealed that the diversity of this locus was drastically reduced in the cultivated species [26, 55]. Only two SNPs have been identified in this gene responsible for the large-fruited phenotype, which are the same two SNPs that we have found in our population. On the other hand, the GWAS analysis for fruit weight revealed an associated genomic region on chromosome 2 between 50.51 and 50.55 Mb in the region where the *FW2.2* gene is located [27]. Similarly, but not as precisely as in our ToMAGIC populations, in the tomato MAGIC developed by Pascual *et al.* [17], a peak with the highest LOD value between 46.35 and 47.49 Mb was also identified. The *FW2.2* gene is responsible for up to 30% of the fruit weight variation between large domesticated tomatoes and the small-fruited wild relatives [56]. All modern tomatoes contain the large-fruited allele for *FW2.2* [21, 57], which was also identified in the two large-fruited SLC ToMAGIC founders. Molecular evolutionary studies suggested that this allele originated in wild tomatoes long before the process of domestication [19]. Indeed, fruit weight was strongly selected in SLC in the Andean region of Ecuador and Northern Peru prior to the domestication of tomato in Mesoamerica [21].

Anthocyanins are mainly responsible for purple pigmentation in tomato leaf veins, leaf tissues, and stem [58, 59]. Plant anthocyanins are more commonly present in wild tomato species, where they have a main protective function against ultraviolet (UV)–visible light and other stressful conditions such as cold temperature, pathogens, or drought [60–62]. The GWAS results identified an associated genomic region, which colocalized with the previously described *SlMYB-ATV* gene. Overexpression of the coding protein acts as an inhibitor of anthocyanin production by silencing key regulators of the biosynthesis pathway [28, 63]). The *atv* mutation was described as a 4-bp insertion in the second exon, which led to a frameshift variant resulting in a premature stop codon with a strong impact in the polypeptide. This mutation was identified as the causal agent of anthocyanin production in the vegetative part of the plant [28]. Here, a novel mutation in the “purple” SP4 founder was found. Specifically, a 9-bp deletion leading to a disruptive inframe deletion, which directly affects the transcription repressor MYB domain, was identified. Future experiments could confirm whether this deletion influences the transcription or subcellular activity of *SlMYB-ATV* by transient expression assays. This demonstrates the significance of the ToMAGIC population as a reservoir of novel candidate genes and causative alleles. Interestingly, of the four SP founders, SP4 is the only one showing anthocyanin pigmentation as well as the one collected at the highest altitude (1020 m) and lowest mean annual temperature (13°C), in agreement with the proposed role of anthocyanins as UV sunscreens in cold temperatures [24].

Cultivated tomato leaf morphology has typical bipinnate compound leaves with moderately deep lobes, while there is a huge diversity of leaf morphology among wild tomato species ( [35, 64, 65]. Since leaf lobing/serration and leaf complexity traits are correlated, both traits have usually been studied together [64]. Actually, the GWAS results identified an associated genomic region on chromosome 4 around 62 Mb position for both traits, and candidate genes affecting both traits were identified within this genomic region. Although the *AP3/DEF* gene has mainly been related to petal and sepal development, other genes belonging to the same MADS box family are involved in tomato leaf development. Specifically, the *APETALA1/FRUITFULL* (*AP1/FUL*) MADS box genes are involved in the organogenic activity of the leaf margin and leaf complexity [66]. The *ANT* gene also belongs to a family of APETALA 2/ETHYLENE RESPONSE FACTOR (AP2/ERF) domain transcription factors, which affects plant leaf shape and size by regulating cell proliferation [33]. The *OVATE* gene was first identified in tomato as a key regulator of fruit shape [67]. However, expression of *OVATE* genes can also result in dwarf plants with shorter and thicker organs such as rounder leaves [32]. The tomato *KNOTTED1* promotes cytokinin biosynthesis, which is directly related to cell proliferation [65], and different levels of cytokinins led to a broad spectrum in leaf complexity [34, 68]. This gene has a key role in the molecular mechanism behind leaf development and evolution and has been repeatedly exploited to generate natural variations in leaf shape). The *IAA9* gene is a transcriptional repressor in auxin signal transduction [69]. Tomato mutants for *IAA9* also showed altered leaf morphology with the compound leaf changing to a single leaf [35, 69, 70]. In this way, leaf development is mainly influenced by cell proliferation and different hormones as a result of the activity of a complex gene network [65]. An accurate phenotyping of the ToMAGIC population for these traits has allowed to narrow down a genomic region that harbours a large number of genes related to leaf morphology. This genomic region could be further narrowed down by studying the segregation of the cross between two isolines to enable the identification of the responsible gene/s.

The existence of early flowering alleles in wild species indicates the relevance of exploiting the genetic variation present in tomato wild relatives [71]. Although the mechanisms controlling the transition from vegetative to reproductive growth are complex, several genes involved in flowering regulation are known [72, 73]. The number of leaves below the first inflorescence trait is a proxy for earliness in tomato [74] and is easily scored and commonly assessed to evaluate the earliness in tomato [71, 75, 76]. The GWAS analysis for the number of leaves below the first inflorescence identified an association on chromosome 11, where several genes related to flowering time were found (two *FT* genes, *SP1*, and *J*). The most studied *FT* gene is the tomato ortholog *SINGLE-FLOWER TRUSS* (*SFT*) gene on chromosome 3, which encodes for florigen and induces flowering in day-neutral [72, 73, 77]. Here, we report the *FT1* gene on chromosome 11, a paralogue of the *SFT* gene, which may also be involved in the flowering regulation. The *SP1* gene is a member of the *CETS* family of regulatory genes, together with *FT* genes, controlling flowering time [37]. However, they play an antagonistic role, since *SP1* delays flowering in tomato [73]. The *J* gene is involved in the same pathway as the *SFT* gene but with a small role in flowering promotion [38, 73]. A better understanding of the mechanisms underlying the tomato flowering regulatory pathways will allow breeding to target more precise candidate genes for the induction of early flowering. Nevertheless, once again, the ToMAGIC population has led us to a genomic region directly involved in the transition to flowering, pointing to new candidate genes.

Overall, the genotyping results together with the large morphological variation observed in the new interspecific SLC/SP tomato MAGIC population, as well as the appearance of transgressive phenotypes, indicate that recombination and variation were maximized in the final population. The ToMAGIC population size was suitable for an accurate association detection of well-known traits as a proof of concept to validate the efficiency of the population. The ToMAGIC population has demonstrated a high potential for the fine mapping of traits of interest from different plant parts. A novel mutation was identified in the *SlMYB-ATV* gene responsible of the anthocyanin pigmentation in vegetative tissues. Further transcriptional expression analysis of genes under the anthocyanin biosynthesis pathway and gene editing will be essential to elucidate the effect of this mutation. Candidate genes were proposed for leaf morphology and earliness related traits. Fine mapping and further gene expression analysis could provide deeper insights into the genetic control of these traits. Given the fact that the population contains representatives of the tomato ancestor (SP) and the primitive weedy forms (SLC) of tomato, it can also be a tool of great relevance for studying the genetic changes in the early stages of tomato domestication. It is also evident from our study that the derived ToMAGIC population or core collections developed from it can contribute to tomato genetics research and breeding programmes. Currently, the ToMAGIC population is being assessed for nitrogen use efficiency, drought tolerance, and resistance to different pathogens. Recombinant lines with combinations of traits of interest present in different founders can also be of direct interest to breeders or even for selection of small-fruited new cultivars.

## Materials and methods

### ToMAGIC founders

The interspecific tomato MAGIC (ToMAGIC) population was developed through the intercrossing of SLC and SP accessions. Founders consist of four weedy SLC, i.e., BGV007931 (SLC1), LA2251 (SLC2), PI487625 (SLC3), and BGV006769 (SLC4), and four wild SP*,* i.e., BGV007145 (SP1), BGV006454 (SP2), BGV015382 (SP3), and BGV013720 (SP4). Their geographical origin, including geographical coordinates and altitude, and environmental parameters (mean temperature, temperature range, precipitation, etc.) are known [24]. With respect to the Heinz 1706 SL4.0 reference genome [78], the total variants identified in SLC accessions ranged from 1.2 million in SLC2 to 1.9 million in SLC1, while in the SP accessions, they ranged from 3.1 million in SP4 to 4.8 million in SP3 [22]. This set of variants was over 1600-fold more abundant than the one used in the previous study of Blanca *et al.* [21], where the eight founders were also genotyped.

### ToMAGIC population development

Although low heterozygosity levels were observed for founders in previous studies [21], before starting with the ToMAGIC population cross-design, two generations of selfing of the founders were performed to ensure high homozygosity. To develop the ToMAGIC population, founder lines were intercrossed by following a “funnel” approach including two extra generations of intercrosses among the offspring of the double hybrid crosses. These extra steps were performed to increase recombination events among the genomes of the eight founders during the population development to achieve better mapping and QTL identification resolution [14]. The first step is developing the MAGIC population consistent in crossing the SLC parents with the SP ones to produce interspecific F_1_ hybrids (SLC1 × SP2, SLC2 × SP1, SLC3 × SP4, and SLC4 × SP3). These F_1_ hybrids were subsequently intercrossed in pairs (SLC1 × SP2 with SLC2 × SP1 and SLC3 × SP4 with SLC4 × SP3) directly (′) and reciprocally (″) to obtain four genetically segregating double hybrids (DHY1′, DHY1″, DHY2′, and DHY2″). In this way, genomes from both species were mixed since the beginning of the development of the MAGIC population. Then, DHY1′ or DHY1″ individuals were crossed with DHY2′ or DHY2″ individuals, obtaining a set of quadruple hybrids coming from the first intercross generation (IC1), which were an admixture of the genomes of the eight founders. DHYs were crossed by following a chain pollination scheme, where each individual was used as a female and male parent of different crosses [44, 79]. Initially, the first DHY1 line served as the male parent in an intercross with the first DHY2 line, which acted as the female parent. This pattern continued with the roles reversed: the first DHY2 line then served as the male parent in an intercross with the second DHY1 line as the female. This alternating pattern persisted until the final stage, where the last DHY2 line was used as the male parent in an intercross with the first DHY1 line, again acting as the female (Fig. S8). In the same way, individuals from the second intercross (IC2) generation were also intercrossed following a chain pollination scheme. This step was repeated to obtain the individuals from the third intercross generation (IC3). Finally, progenies of the IC3 were selfed for five generations by SSD to obtain the ToMAGIC recombinant inbred lines. To accelerate the obtention of the SSD generations, selfings were stimulated by mechanical vibration and pruning was done manually, regulating vegetative growth and flowering. A set of 354 ToMAGIC lines were used in this study for phenotyping and genotyping.

Seeds from the 354 ToMAGIC lines were germinated in seedling trays with Humin-substrat N3 substrate (Klasmann-Deilmann, Germany) in a climatic chamber under a photoperiod and temperature regime of 16 h light (25°C) and 8 h dark (18°C). Plantlets were subsequently transplanted to individual thermoformed pots (1.3-l capacity) for acclimatization and grown in a pollinator-free glasshouse of the Universitat Politècnica de València (Valencia, Spain). Plants were fertirrigated using a drip irrigation system and trained with vertical strings. Phytosanitary treatments against whiteflies and *Tuta absoluta* were performed when necessary.

### High-throughput genotyping

Young leaf tissue was sampled from the 354 ToMAGIC lines. Genomic DNA was extracted using the SILEX extraction method [80]. DNA quality and integrity were checked by agarose electrophoresis and NanoDrop ratios (260/280 and 260/230), while its concentration was estimated using a fluorescent DNA intercalating agent (e.g., Quant-iT PicoGreen dsDNA Assay Kit, Thermo Fisher Cat. No. P7589) and a microplate reader (Thermo Fisher Scientific). Samples were sent to IGATech company (Udine, Italy) for library preparation and sequencing (150 paired-end) for a high-throughput genotyping using a newly developed 12k probes tomato SPET panel, which is considerably improved over the original 5 k probes tomato set [42]. The new SPET panel comprises 12 000 probes and was developed by selecting the most informative and reliable polymorphisms (of which ~11 500 within 100 nt of a gene and ~500 in intergenic regions) (Aprea *et al*., in preparation).

Cleaning of raw reads was performed using Fastp [81]. Clean reads were mapped onto the tomato reference genome Heinz 1706 SL4.0 [78] using BWA-MEM [82] with default parameters; finally, GATK was used for variant calling [83], following the best practices recommended by the Broad Institute. The SNPs identified by the tomato SPET panel were first filtered by coverage ≥10 and quality GQ ≥20, removed the monomorphic sites using bcftools [84], and then filtered using the TASSEL software (ver. 5.0, [85]) to retain the most reliable ones (minor allele frequency >0.01, missing data <0.1, and maximum marker heterozygosity <0.7). In addition, a LD k-nearest neighbour genotype imputation method (LD KNNi) was performed to fill the missing calls or genotyping gaps [86]. Final marker density along chromosomes was represented using the R package chromPlot [87].

### Population diversity analysis

A PCA was performed to assess the population structure of the MAGIC population. PCA scores were generated in TASSEL software (ver. 5.0, [85]). For graphically plotting the final PCA results, the R package ggplot2 was used [88]. A heat map of the kinship matrix to identify possible relationships between lines was generated with GAPIT software (v.3, [89]). A dendrogram of the MAGIC population was generated using the neighbour-joining method [90], and the graphical representation was displayed and edited using the iTOL v.4 software [91] to evaluate the genetic similarities among ToMAGIC lines and founders. Parental contribution to the ToMAGIC lines and haplotype blocks was estimated by using the R package HaploBlocker [92].

### ToMAGIC phenotyping

A proof of concept for testing the potential of the MAGIC population for GWAS analysis and detection of genomic regions associated with different types of traits was performed by phenotyping the eight parents and the 354 ToMAGIC lines for a set of traits from different plant organs. The traits evaluated included two related to fruit size (fruit locule number and fruit weight), one to plant pigmentation (plant anthocyanin), two to leaf morphology (lobing/serration and leaf complexity), and one to earliness (number of leaves below the first inflorescence). Tomato fruits were evaluated for fruit weight and cut transversally for locule number counting. Presence of plant anthocyanin was observed in vegetative plant parts (stem, branches, leaf veins, or leaf area) and scored in a range from 0 (slight presence, mainly on the stem) to 4 (strong presence in all plant parts). Leaf lobing/serration was scored in a range from 1 (lack of lobing/serration) to 7 (very serrated leaf). Leaf complexity was screened using a binary classification for pinnate (0) and bipinnate (1) compound leaves. The number of leaves below the first inflorescence was recorded by counting the leaves of the primary shoot when the first flower bud was visible. Pearson pair-wise coefficient of correlation (*r*) among traits was calculated, and their significance was assessed using a Bonferroni correction at the *P* < 0.05 probability level [93] using R packages psych [94] and corrplot [95].

### Genome-wide association study

Using the genotypic and phenotypic data collected from the ToMAGIC lines, GWAS analyses were performed for the selected traits using the GAPIT software (v.3, [89]). General linear model (GLM), mixed linear model (MLM), and BLINK analyses were conducted for the association study [46–48]. Comparison of models was displayed in roundness Manhattan plots. The multiple testing was corrected with the Bonferroni and the FDR methods [96, 97] with a significance level of 0.05 [98]. Bonferroni threshold is defined as the −log10 of the desired overall alpha level (*α* = 0.05) divided by the total number of SNPs. Therefore, it remains constant among the different association models. Meanwhile, FDR threshold values are retrieved by adjusting *P* values to control the proportion of false positive. Thus, FDR thresholds vary among models and traits. SNPs with a limit of detection (LOD) score (calculated as −log10[*P* value]) exceeding these specified thresholds or cutoff values in the three GWAS models were considered significantly associated with the traits under evaluation. Associations were considered significant if the same SNP exceeded the cut-off thresholds in at least two of the implemented models, indicating robustness. The top significant SNPs delimited the candidate genomic regions. All markers within these genomic regions were used to calculate the correlation coefficient (*r*^2^). SNPs with default *r*^2^ values greater than 0.5 were considered for haplotype block estimation. The R package geneHapR was used for haplotype statistics [99]. The genes underlying the haplotype blocks were retrieved from the Heinz 1706 SL4.0 tomato reference genome [78]. Candidate genes were assessed by SnpEff software v 4.2 prediction [29] of the eight MAGIC founders’ resequencing data [100] in order to identify causative mutations contrasting for different phenotypes. The Integrative Genomics Viewer tool was used for the visual exploration of founder genome sequences to validate SnpEff results [101]. A conservative domain analysis was performed using the NCBI conserved domain server (https://www.ncbi.nlm.nih.gov/Structure/cdd/wrpsb.cgi) to assess the predicted variants at the protein level. The BLASTp (e-value cut-off of 1e^−10^) alignment tool and EnsemblPlants browser were used to compare the homology of protein sequences encoded by genes belonging to the same gene family. Haplotype and phenotype boxplots and density plots were generated with the R package ggplot2 [88]. To assess the significance of differences among different haplotypes, pairwise *t*-tests were performed. Density plots represent the density of the data at each value of *x*, allowing peak values to potentially exceed 1, particularly when data are densely concentrated around specific values.

## Acknowledgements

This work was supported by grant PID2020-118627RB-I00 funded by MICIU/AEI/10.13039/501100011033, TED2021-129296B-I00 funded by MCIN/AEI/10.13039/501100011033 and European Union Europea NextGenerationEU/PRTR, CIPROM/2021/020 funded by Conselleria d’Innovació, Universitats, Ciència i Societat Digital of the Generalitat Valenciana, the HARNESSTOM innovation action (grant agreement no. 101000716) funded by the European Commission H2020 Research and Innovation Programme, and the Horizon Europe PRO-GRACE project (grant agreement no. 10194738). Andrea Arrones is grateful to Spanish Ministerio de Ciencia, Innovación y Universidades for a predoctoral (FPU18/01742) contract. Oussama Antar is grateful to Conselleria d'Innovació, Ciència i Societat Digital of the Generalitat Valenciana for a predoctoral grant within the Santiago Grisolía program (CIGRIS/2021/113). Leandro Pereira-Dias is grateful to Universitat Politècnica de Valencia and the Spanish Ministerio de Universidades for a post-doctoral grant under the Margarita Salas funded by the European Union NextGenerationEU/PRTR. Andrea Solana is grateful to Spanish Ministerio de Ciencia e Innovación for a predoctoral grant (PRE2022-102368) funded by MCIN/AEI/10.13039/501100011033 and FSE+. Pietro Gramazio is grateful to Spanish Ministerio de Ciencia e Innovación for a post-doctoral grant (RYC2021-031999-I) funded by MCIN/AEI/10.13039/501100011033 and the European Union through NextGenerationEU/PRTR. Funding for open access: Universitat Politècnica de València.

## Author contributions

S.V., P.G., M.J.D., and J.P. conceived the idea and supervised the manuscript; A.A., O.A., L.P.-D., A.S., and M.J.D. performed the field trials. G.A. and G.G. in collaboration with TECAN Genomics designed the 12k SPET panel. All authors analysed the results. A.A. and O.A. prepared a first draft of the manuscript, and the rest of the authors reviewed and edited the manuscript. All authors have read and agreed to the published version of the manuscript.

## Data availability

The datasets presented in this study can be found in online repositories. The names of the repository/repositories and accession number(s) can be found below: https://www.ncbi.nlm.nih.gov/, PRJNA616074 (founders resequencing data), and PRJNA1103671 (ToMAGIC lines genotyping).

## Conflict of interest statement

The authors declare that the research was conducted in the absence of any commercial or financial relationships that could be construed as a potential conflict of interest.

## Supplementary Data

Supplementary data is available at Horticulture Research online.

## Supplementary Material

Web_Material_uhae154
