# Supplementary figures and images for: A novel tomato interspecific (Solanum lycopersicum var. cerasiforme and Solanum pimpinellifolium) MAGIC population facilitates trait association and candidate gene discovery in untapped exotic germplasm
Source: Hortic Res. 2024 Jun 3;11(7):uhae154. doi: 10.1093/hr/uhae154 (PMC11246243; doi:10.1093/hr/uhae154)

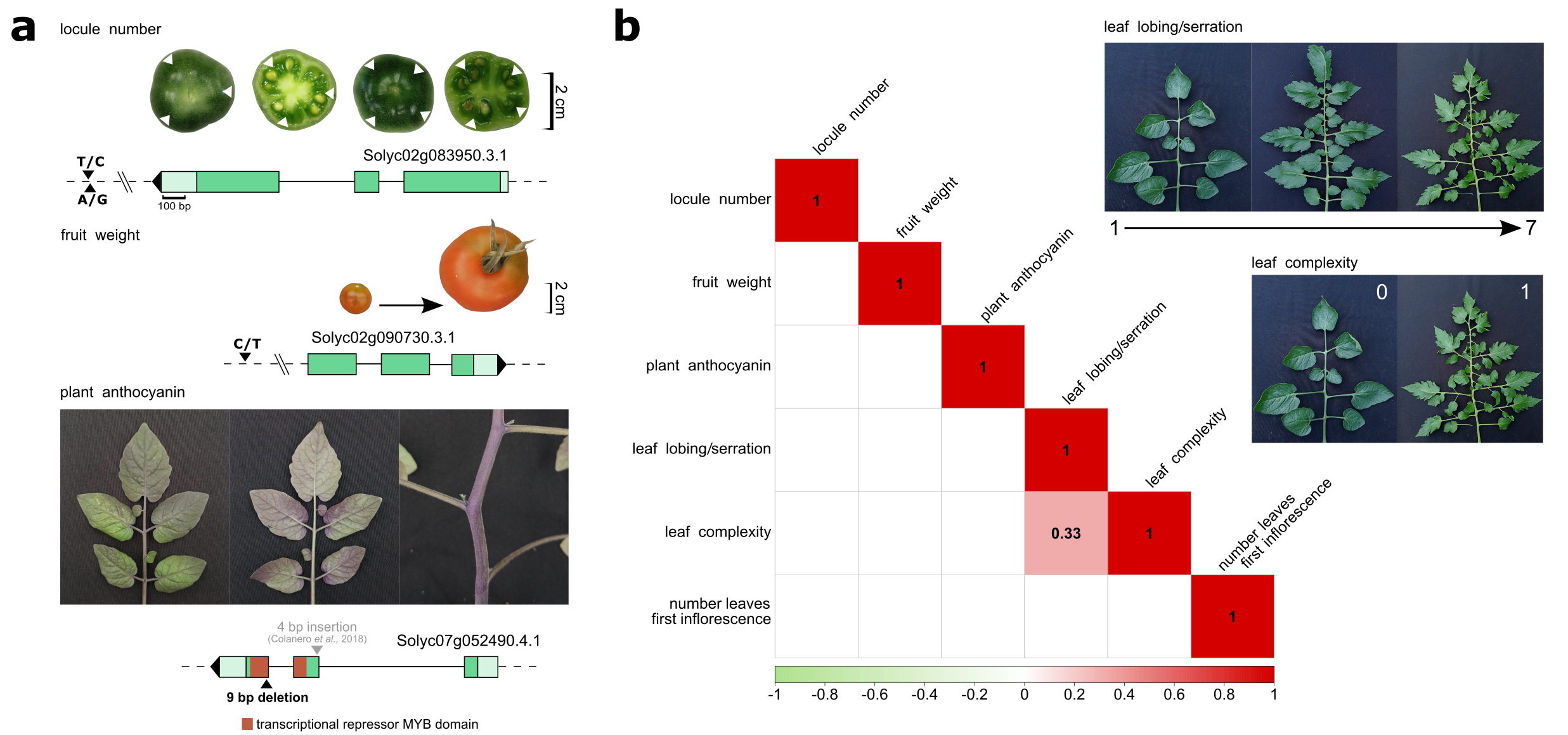

Supplement: Web_Material_uhae154 [file web_material_uhae154.zip › Supplementary Figure 2..tif]
